# Correlação de Imagem Espaço-Temporal: Imagens Tridimensionais para Triagem Cardíaca Fetal e Avaliação de Doenças Cardíacas Congênitas

**DOI:** 10.36660/abc.20230580

**Published:** 2024-04-15

**Authors:** André Malho, Renato Silveira Ximenes, Nathalie Jeanne Bravo-Valenzuela, Edward Araujo

**Affiliations:** 1 Fundação Latino-Americana de Medicina Fetal Campinas SP Brasil Fundação Latino-Americana de Medicina Fetal (FMF-LA), Campinas, SP – Brasil; 2 Universidade Federal de São Paulo Escola Paulista de Medicina Departamento de Obstetrícia São Paulo SP Brasil Departamento de Obstetrícia – Escola Paulista de Medicina – Universidade Federal de São Paulo (EPM-UNIFESP), São Paulo, SP – Brasil; 3 Universidade Federal do Rio de Janeiro Cardiologia Pediátrica Departamento de Pediatria Rio de Janeiro RJ Brasil Departamento de Pediatria – Cardiologia Pediátrica – Universidade Federal do Rio de Janeiro (UFRJ), Rio de Janeiro, RJ – Brasil; 4 Universidade Municipal de São Caetano do Sul Disciplina de Saúde da Mulher São Caetano do Sul SP Brasil Disciplina de Saúde da Mulher – Universidade Municipal de São Caetano do Sul (USCS), São Caetano do Sul, SP – Brasil

**Keywords:** Coração Fetal, Cardiopatias Congênitas, Ultrassonografia, Imageamento Tridimensional

Os defeitos cardíacos estão entre as malformações congênitas mais comuns, ocorrendo em 4 a 12 para cada 1.000 nascidos vivos, dependendo da população estudada. A doença cardíaca congênita (DCC) é uma causa significativa de mortalidade relacionada a defeitos congênitos.^1^ Portanto, o diagnóstico pré-natal de DCC é fundamental para melhorar o prognóstico associado a esta doença. Nesse cenário, o desenvolvimento de programas de triagem ultrassonográfica cardíaca pré-natal são ferramentas importantes que têm aumentado a detecção precoce de DCC. Esses programas tornam isso possível ao adicionar a via de saída ventricular e as incidências do mediastino superior (três vasos - 3V e 3V com traqueia) ao padrão de visualização de quatro câmaras (4C) durante a varredura do ultrassom cardíaco.^2^

O desenvolvimento de tecnologias avançadas de imagem, como ultrassonografia tridimensional e quadridimensional (3D/4D) e correlação de imagens espaço-temporais (STIC), tem melhorado a qualidade das imagens ultrassonográficas do coração fetal com impacto positivo sobre o diagnóstico de malformações cardíacas. A tecnologia 4D-STIC permite a captura de milhares de imagens cardíacas usando um transdutor volumétrico 3D, realizando uma única varredura em câmera lenta com duração de 7,5 a 15 segundos da visualização 4C do coração fetal. Os volumes 3D/4D adquiridos contêm um "bloco" de informações do ciclo cardíaco completo, que pode ser armazenado para posterior navegação off-line do coração fetal, permitindo uma análise mais detalhada da anatomia cardíaca pelo ultrassonografista, ou enviado via link da Internet para análise por especialistas em centros terciários.^3^

O software denominado HDlive é uma técnica de desenho de superfície que se diferencia dos métodos convencionais de renderização 3D por oferecer ao operador a capacidade de selecionar livremente a fonte de luz sob qualquer ângulo, proporcionando sensações realistas das estruturas cardíacas e seus vasos, e permitindo a análise dos detalhes anatômicos do coração fetal.^4^ Neste artigo, descrevemos alguns exemplos de imagem de DCC diagnosticada pela adição de HDlive e HDlive Flow Silhouette ao ultrassom 3D/4D com STIC.

**Figura 1:**
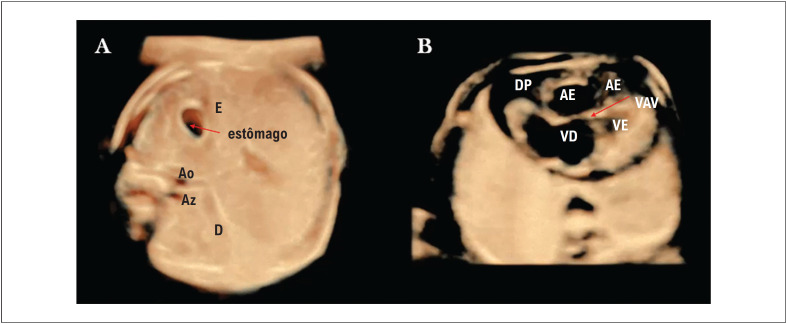
Ultrassonografia tridimensional com STIC e HDlive em feto (23 semanas e 6 dias) com isomerismo atrial esquerdo, dextrocardia, defeito do septo atrioventricular completo (DSAV) e bloqueio atrioventricular completo (BAVT). A) O isomerismo atrial esquerdo, facilmente reconhecido pelo fato do vaso venoso (ázigo) ser posterior ao vaso arterial (aorta) na vista abdominal superior. B) DSAV completamente desequilibrado pode ser reconhecido por uma visão anormal das quatro câmaras: observe a válvula atrioventricular comum com um único orifício (seta vermelha) e os ventrículos de tamanhos desiguais. Observa-se derrame pericárdico (DP) devido ao bloqueio AV completo. AE: átrio esquerdo; VAV: válvula atrioventricular; Ao: aorta; Az: ázigo; E: lado esquerdo; D: lado direito.

O exame ultrassonográfico da parte superior do abdômen do feto permite uma avaliação do local. Em sua posição normal (situs solitus), o vaso arterial (aorta) estará localizado à esquerda e atrás da veia cava inferior. Na vista abdominal superior, o isomerismo esquerdo é facilmente reconhecido pelo fato de o vaso venoso (ázigo/hemiázigo) ser posterior ao vaso arterial (aorta). O isomerismo esquerdo (situs ambíguo) é uma condição na qual os átrios e os pulmões estão duplicados no lado esquerdo. Em quase todos os casos de isomerismo à esquerda, o segmento hepático da veia cava inferior, com drenagem pelas veias ázigos e/ou hemiázigos, está ausente ( Figura 1A ). Além disso, como ambos os átrios são morfologicamente esquerdos, o nó sinoatrial direito está ausente e, consequentemente, o risco de bradicardia devido ao bloqueio cardíaco aumenta no isomerismo esquerdo. Na realidade, DCCs mais complexas, como defeito do septo atrioventricular (DSAV) desequilibrado e via de saída dupla do ventrículo direito, geralmente estão associadas a anormalidades de situs ( Figura 1B ).^4 , 5^

**Figura 2:**
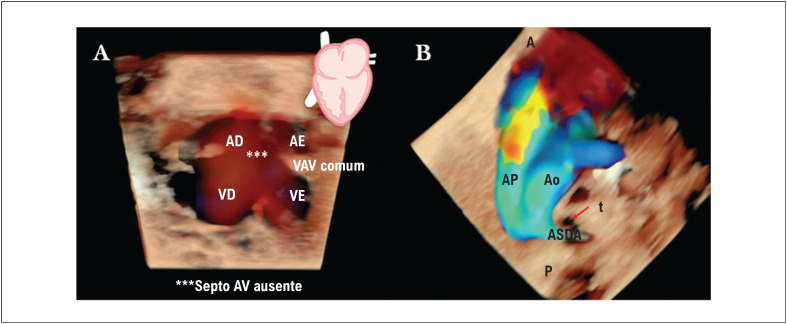
Observe as imagens cardíacas mais realistas com ultrassom tridimensional em modo HDlive Silhouette de um feto (30 semanas e 6 dias) com trissomia do 21. A) Durante a diástole com HDlive flow no corte de quatro câmaras, o sinal em "H" da válvula atrioventricular (AV) comum (***) é uma pista importante para o diagnóstico de defeito do septo atrioventricular completo (DSAV). B) Vista de três vasos e traqueia (3VT) mostrando ASDA com trajeto retrotraqueal no mesmo feto. VE: ventrículo esquerdo; AE: átrio esquerdo; AD: átrio direito; VD: ventrículo direito; VAV comum: válvula atrioventricular comum com orifício único; Ao: aorta; AP: artéria pulmonar; ASDA: artéria subclávia direita aberrante; t: traqueia (seta vermelha); A: anterior; P: posterior.

O DSAV é uma DCC que resulta da falha na fusão do septo atrioventricular. A forma clássica completa do DSAV é caracterizada por uma válvula atrioventricular comum, defeito septal atrial *ostium primum* e uma comunicação interventricular de entrada. Por meio do Doppler colorido, esse diagnóstico é facilmente reconhecido na incidência 4C do coração fetal pelo sinal em "H" devido à ausência do septo atrioventricular ( Figura 2 ). Além disso, imagens anatomicamente realistas da válvula atrioventricular comum, com detalhes de sua inserção, podem ser obtidas usando ultrassom 3D/4D e HDlive. O DSAV com hipoplasia de um ventrículo é referido como "desequilibrado" e está tipicamente associado à síndrome heterotaxia (isomerismo).^4 , 5^ O DSAV completo tem forte associação com malformações extracardíacas e síndromes como a trissomia do 21. Semelhante ao DSAV, a artéria subclávia direita aberrante (ASDA) foi descrita como um marcador para anomalias cromossômicas, como a trissomia do 21. A ASDA surge como um quarto vaso da aorta e não da artéria braquiocefálica. Seu curso é posterior à traqueia, não anterior como nos corações normais ( Figuras 2B e 3 ). Assim, o cariótipo fetal deve ser discutido com os pais quando tais diagnósticos são feitos.^4 , 5^

**Figura 3:**
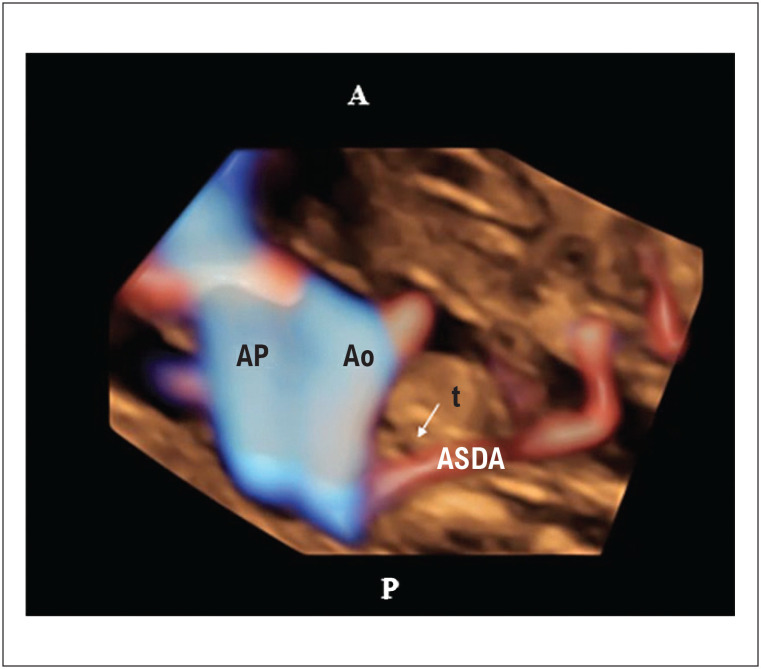
Ultrassonografia tridimensional com HDlive flow e modos Silhouette da visualização de três vasos e traqueia (3VT) mostrando uma artéria subclávia direita aberrante (ASDA) de um feto com 20 semanas e 6 dias. A ASDA surge da aorta como o quarto vaso. Foi descrita como um marcador de risco para anomalias cromossômicas, como a trissomia do 21. Observa-se que a ASDA passa posteriormente à traqueia na visualização 3VT. A artéria subclávia direita normal é um vaso em forma de S que passa anteriormente à traqueia. Ao: aorta; AP: artéria pulmonar; ASDA: artéria subclávia direita aberrante, t: traqueia; P: posterior; A: anterior.

**Figura 4:**
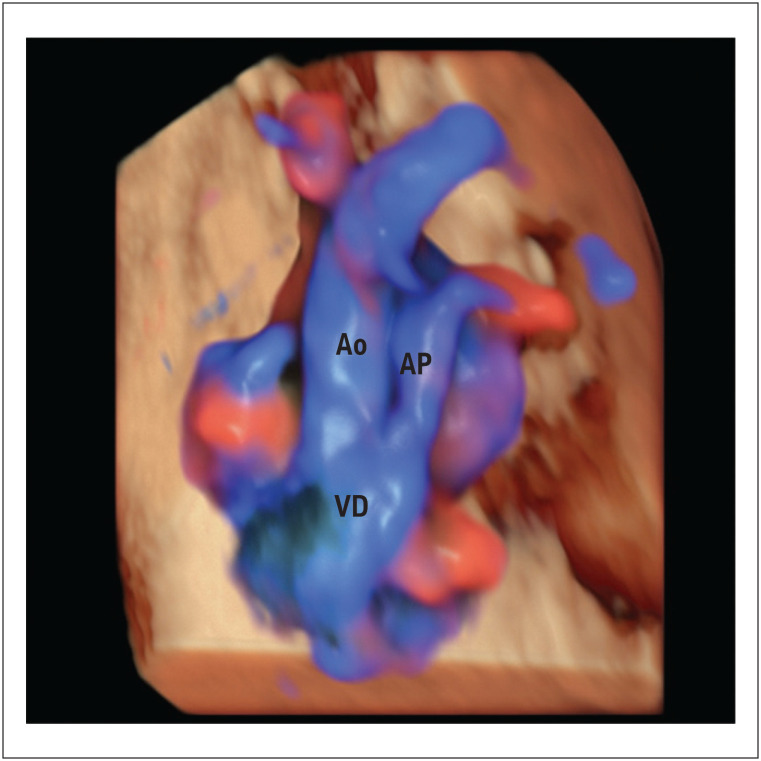
Saída dupla do ventrículo direito tipo Fallot em feto com 29 semanas e 3 dias. Vistas anormais da via de saída com HDlive flow e modos Silhouette, mostrando as grandes artérias originadas do ventrículo direito com estenose pulmonar. Observa-se uma estenose pulmonar. Observa-se uma artéria pulmonar pequena (AP < Ao). VD: ventrículo direito; Ao: aorta; AP: artéria pulmonar.

A dupla via de saída do ventrículo direito (DVSVD) refere-se a um grupo de DCCs em que ambas as grandes artérias se originam predominantemente (> 50%) do ventrículo morfologicamente direito. A DVSVD é descrita como tipo 1- Fallot, quando há estenose pulmonar ( Figura 4 ), tipo 2- Taussig-Bing, quando as grandes artérias estão em relação paralela ("grandes artérias transpostas"), e do tipo defeito do septo ventricular quando as grandes artérias estão normalmente relacionadas sem estenose pulmonar. A presença de um septo ventricular desalinhado e a descontinuidade aórtico-mitral nas incidências da via de saída ventricular são fundamentais para esse diagnóstico pela ecocardiografia.^4 , 5^

Conclui-se que as tecnologias avançadas de ultrassonografia cardíaca fetal em 3D/4D, quando disponíveis, podem melhorar o rastreamento e o diagnóstico de doença coronariana, permitindo uma análise da anatomia cardíaca mais detalhada com imagens mais realistas.
