# Supplementary material for: Correlação de Imagem Espaço-Temporal: Imagens Tridimensionais para Triagem Cardíaca Fetal e Avaliação de Doenças Cardíacas Congênitas
Source: Arq Bras Cardiol. 2024 Apr 15;121(4):e20230580. [Article in Portuguese] doi: 10.36660/abc.20230580 (PMC11081425; doi:10.36660/abc.20230580)
Supplement: Translation

# Translation: Spatio-Temporal Image Correlation: Three-Dimensional Imaging for Fetal Cardiac Screening and Congenital Heart Disease Assessment

**DOI:** 10.36660/abc.20230580i

**Authors:** André Malho, Renato Silveira Ximenes, Nathalie Jeanne Bravo-Valenzuela, Edward Araujo

**Affiliations:** 1 Campinas SP Brazil Fundação Latino-Americana de Medicina Fetal (FMF-LA), Campinas, SP – Brazil; 2 São Paulo SP Brazil Departamento de Obstetrícia – Escola Paulista de Medicina – Universidade Federal de São Paulo (EPM-UNIFESP), São Paulo, SP – Brazil; 3 Rio de Janeiro RJ Brazil Departamento de Pediatria – Cardiologia Pediátrica – Universidade Federal do Rio de Janeiro (UFRJ), Rio de Janeiro, RJ – Brazil; 4 São Caetano do Sul SP Brazil Disciplina de Saúde da Mulher – Universidade Municipal de São Caetano do Sul (USCS), São Caetano do Sul, SP – Brazil

**Keywords:** Fetal Heart, Congenital Heart Defects, Ultrasonography, Three-Dimensional Imaging

Heart defects are among the most common congenital malformations, occurring in 4 to 12 per 1,000 live births, depending on the population studied. Congenital heart disease (CHD) is an important cause of mortality related to birth defects.^1^Therefore, prenatal diagnosis of CHD is crucial to improve the prognosis associated with this disease. In this scenario, the development of prenatal cardiac ultrasound screening programs serves as crucial tools that have increased the early detection of CHD. These programs achieve this by adding the ventricular outflow tract and superior mediastinum views (three vessels - 3V and 3V with trachea) to the standard four-chamber (4C) view when scanning the cardiac ultrasound.^2^

The development of advanced imaging technologies, such as three- and four-dimensional (3D/4D) ultrasound and Spatio-Temporal Image Correlation (STIC), has improved the quality of ultrasound images of the fetal heart with a positive impact on the diagnosis of cardiac malformations. The 4D-STIC technology allows the capture of thousands of cardiac images using a 3D volumetric transducer by a single 7.5 to 15-second slow-motion scan of the 4C view of the fetal heart. The acquired 3D/4D volumes contain a "block" of information of the complete cardiac cycle that can be stored for later off-line navigation of the fetal heart, allowing detailed analysis of cardiac anatomy by the sonographer, or sent via Internet link for analysis by experts in tertiary centers.^3^

The software called HDlive is a technique of surface drawing that differs from conventional 3D rendering methods because the operator can freely select the light source at any angle, providing realistic sensations of cardiac structures and their vessels, allowing the analysis of anatomical details of the fetal heart.^4^In this article, we describe some imaging examples of CHD diagnosed by adding HDlive and HDlive Flow Silhouette to 3D/4D ultrasound with STIC.

Ultrasound examination of the upper abdomen of the fetus provides a situs assessment. If the situs is normal (situs solitus), the arterial vessel (aorta) is located to the left and behind the venous vessel, which is the inferior vena cava. In the upper abdominal view, left isomerism is easily recognized by the fact that the venous vessel (azygos/hemiazygos) is posterior to the arterial vessel (aorta). Left isomerism (situs ambiguous) is a condition in which the atria and lungs are duplicated on the left side. In almost all cases of left isomerism, the hepatic segment of the inferior vena cava with drainage via the azygous and/or hemiazygous veins is absent ( Figure 1A ). Furthermore, since both atria are morphologically left-sided, the right sinoatrial node is absent and consequently the risk of bradycardia due to heart block is increased in left isomerism. In fact, more complex CHDs, such as unbalanced atrioventricular septal defect (AVSD) and double right ventricle outflow tract, are generally associated with situs abnormalities ( Figure 1B ).^4 , 5^

**Figure 1:** Three-dimensional ultrasound with STIC and HDlive in a fetus (23 weeks and 6 days) with left atrial isomerism, dextrocardia, complete atrioventricular septal defect (AVSD) and complete atrioventricular block. A) The left atrial isomerism, which is easily recognized by the fact that the venous vessel (azygous) is posterior to the arterial vessel (aorta) in the upper abdominal view. B) Completely unbalanced AVSD can be recognized by an abnormal four-chamber view: note the common atrioventricular valve with a single orifice (red arrow) and the unequal-sized ventricles. There is a pericardial effusion (PE) due to complete AV block. LA: left atrium; AVV: atrioventricular valve; Ao: aorta; Az: azygous; L: left side; R: right side.

AVSD is a CHD that results from a failure of the atrioventricular septum to fuse. The classic complete form of AVSD is characterized by a common atrioventricular valve, an ostium primum atrial septal defect and an inlet ventricular septal defect. Using color Doppler, this diagnosis is easily recognized in the 4C view of the fetal heart by an "H-shaped" sign due to the absence of the atrioventricular septum ( Figure 2 ). In addition, anatomically realistic images of the common atrioventricular valve with details of its attachment can be obtained using 3D/4D ultrasound and HDlive. AVSD with hypoplasia of one ventricle is termed unbalanced and is typically associated with heterotaxy syndrome (isomerism).^4 , 5^Complete AVSD has a strong association with extra-cardiac malformations and syndromes such as trisomy 21. Similar to AVSD, aberrant right subclavian artery (ARSA) has been described as a marker for chromosomal anomalies, such as trisomy 21. The ARSA arises as a fourth vessel from the aorta rather than from the brachiocephalic artery. Its course is posterior to the trachea rather than anterior as in normal hearts ( Figures 2B and 3 ). Therefore, the fetal karyotype should be discussed with the parents when such diagnoses are made.^4 , 5^

**Figure 2:** Note the realistic cardiac images using three-dimensional ultrasound with HDlive Silhouette mode in a fetus (30 weeks and 6 days) with trisomy 21. A) During diastole with HDlive flow in the four-chamber view, the "H-shape" of the common atrioventricular (AV) valve (***) is an important clue to the diagnoses of complete atrioventricular septal defect (AVSD). B) Three-vessels and trachea (3VT) view showing ARSA with retrotracheal course in the same fetus. LV: left ventricle; LA: left atrium; RA: right atrium; RV: right ventricle; common AVV: common atrioventricular valve with unique orifice; Ao: aorta; PA: pulmonary artery; ARSA: aberrant right subclavian artery; t: trachea (red arrow); A: anterior; P: posterior.

**Figure 3:** Three-dimensional ultrasound with HDlive flow and Silhouette modes of the three-vessels and trachea (3VT) view showing an aberrant right subclavian artery (ARSA) of a fetus with 20 weeks and 6 days. The ARSA arises from the aorta as the fourth vessel. It has been described as a risk marker for chromosomal abnormalities, such as trisomy 21. Note the ARSA passing posterior to the trachea in the 3VT view. The normal right subclavian artery is an S-shaped vessel passing anterior to the trachea. Ao: aorta; PA: pulmonary artery; ARSA: aberrant right subclavian artery, t: trachea; P: posterior; A: anterior.

Double outlet of the right ventricle (DORV) refers to a group of CHDs in which both great arteries originate predominantly (> 50%) from the morphologically right ventricle. DORV is described as 1- Fallot type, when there is pulmonary stenosis ( Figure 4 ), 2- Taussig-Bing type, when the great arteries are in a parallel relationship ("transposed great arteries"), and is ventricular septal defect type when the great arteries are normally related without pulmonary stenosis. The presence of a misaligned ventricular septal defect and an aortic-mitral discontinuity in the ventricular outflow tract views are the keys to this diagnosis by echocardiography.^4 , 5^

**Figure 4:** Double outlet of the right ventricle type Fallot in a fetus with 29 weeks and 3 days. Abnormal outflow tract views with HDlive flow and Silhouette modes, showing the great arteries arising from the right ventricle with pulmonary stenosis. There is a pulmonary stenosis. Note that the pulmonary artery is small (PA < Ao). RV: right ventricle; Ao: aorta; PA: pulmonary artery.

In conclusion, advanced 3D/4D ultrasound fetal heart imaging technologies, when available, may improve the screening and diagnosis of CHD by providing detailed cardiac anatomy with more realistic images.
